# ¡Presente!: Affirming Latinx voices within health sciences library scholarship

**DOI:** 10.5195/jmla.2021.1295

**Published:** 2021-10-01

**Authors:** Aidy Weeks, Adela V. Justice, Ruby Nugent, Bredny Rodriguez, Brenda Linares

**Affiliations:** 1 aidybert.weeks@unlv.edu, Assistant Professor and Interim Director, GME Liaison Librarian & Collections Manager, UNLV School of Medicine Library, University of Nevada, Las Vegas, Las Vegas, NV; 2 avjustice@mdanderson.org, Senior Librarian, University of Texas MD Anderson Cancer Center–Houston, Houston, TX; 3 ruby.nugent@unlv.edu, Assistant Professor, Biomedical Science Education Librarian, UNLV School of Medicine Library, University of Nevada, Las Vegas, NV; 4 b5rodriguez@ucsd.edu, Collections Strategist for the Health and Life Sciences, University of California San Diego, La Jolla, CA; 5 blinares@kumc.edu, Health Sciences Librarian, University of Kansas Medical Center, Kansas City, KS

**Keywords:** Latinx, Hispanic, Chicanx, BIPOC, medical librarians, health sciences librarians, library workers, information professions, representation, scholarly pipeline

## Abstract

Increasing diverse author representation within medical librarianship scholarship among BIPOC information professionals is an important endeavor that requires closer examination. This commentary looks to examine the ways in which the profession can support Latinx librarians and library workers in fully participating within the scholarly pipeline by exploring our unique and authentic voices, structural barriers, hesitation and fears, Whiteness in the profession and knowledge production, bias in the peer review process, lack of resources and support, and finally, a call to action.

*Llallámosles gran número de libros …, y porque no tenían cosa en que no hubiese superstición y falsedades del demonio, se los quemamos todos, lo cual sintieron a maravilla y les dio mucha pena* [1].

We found a large number of books … and, as they contained nothing in which were not to be seen as superstition and lies of the devil, we burned them all, which [the Maya] regretted to an amazing degree, and which caused them much affliction [2].

—Spanish Bishop Diego de Landa on burning Mayan text, 1562

## INTRODUCTION

The Mayans, native to portions of Central and South America and ancestors to many modern-day Latinx communities, believed that the setting of the sun symbolized death and that its rising each day, a rebirth. We know this from texts that survived centuries after eradication by the Spanish and were later recognized as important contributions to the study of astronomy. In reflecting this celestial dichotomy, one can look no further than the ebb and flow to affirm Voices of Color. Only after witnessing powerful tragedies like the COVID-19 pandemic and health crisis, as well as the widespread repetition of social injustices, do we seek to appreciate the value of these voices, experiences, and contributions. To be BIPOC (Black, Indigenous, and People of Color), or as in the identities of these coauthors, Chicanx, Borinqueña, Afro-Latinx, Latinx, and Chapina, is a laborious journey of rejection, tokenism, impostor syndrome, feigned recognition, misspelled names, mispronunciations, inappropriate stereotypes, and misunderstood cultural beliefs.

Because part of our identity involves the use of *idioma* (language), for this editorial we take liberty to code switch, which is the ability to fuse together English, Spanish, Spanglish, Portuguese, Creole or Criollo, Indigenous, and colloquial variants across the United States, Mexico, Central America, South America, and the Caribbean. Non-English terms are listed in *italics* and English synonyms in parenthesis. When it comes to the term “Latinx,” we agree with Weeks, Nugent, and Corn's (2020) assertion that the term “embraces an inclusive identification of Hispanic/Latin ethnicity and cultural identity and moves away from the feminine/masculine dichotomy of suffixes ‘-o’ and ‘-a’ … common in Spanish language” [3]. We use this term to address all Hispanic/Latinx identities, intersectionalities, and experiences while also recognizing that not everyone within this community agrees with this identifier.

## *NUESTRA VOZ* (OUR VOICE)

*La voz* (the voice) is one of the most powerful thematic elements in Latinx literature. Generations of Latinx peoples have used this type of storytelling to remember, remind, and retain the thoughts and experiences of those who came before. The oral tradition of *testimonios* (testimonies) has allowed Latinx peoples to connect personal and lived history, to eventually become written truths. It is the rising of *la voz* of silence and representative of the other that empowers us. By wielding *la voz*, our people reclaim and take control of the narrative, establishing our representation in the spaces in which we live and work.

This important cultural practice has also informed the work we do as Latinx health sciences librarians and researchers. Our experience engaging in professional scholarship often leaves us unsupported and isolated, both personally and professionally. It requires us to maintain a constant balance of negotiating our cultural identity and *la familia* (the family) with our role as medical librarians in the profession. As a result, Latinx health sciences librarians find themselves living and working in *las fronterizas* (borderlands). Using our shared *testimonios* brings us together in solidarity while navigating an environment that was not inherently created for us to succeed.

The benefit of incorporating Latinx scholars into the literature allows for other ways of conveying scholarship outside the traditional norms of research and academia. Sharing personal stories amongst colleagues through both formal and informal conversations generates ideas and identifies gaps in the literature. The use of *testimonios* is interwoven into everything we do as medical librarians—our instruction, our research, our work with *la comunidad* (the community), and in our roles in the profession.

## STRUCTURAL BARRIERS

As Latinx library scholars, it is imperative that we become active participants in discovering our voices amid the scholarly landscape. This requires that we recognize the structural barriers in place that have inhibited our thoughts and ideas. It is important to explore the following issues in order to validate the professional obstacles we face and to identify clear solutions:

- Hesitation and fears to write and pursue scholarship opportunities
- Whiteness in library and information science (LIS) and knowledge production
- Bias in the peer review process
- Lack of resources and support

## HESITATION AND FEARS

BIPOC information professionals, and by extension potential Latinx library scholars, often hesitate to write and attempt to publish. There are overwhelming stressors including impostor syndrome, that is, “I don't belong in this role” or “I don't hold the level of expertise needed to write on *X* topic.” In addition, we experience being gaslit when others remark that our ideas are not relevant, not rigorous enough, or not fitting within the scope of the profession's praxis. We fortify our fears and hesitation by insulating ideas with tremendous potential through self-doubt and repeated questioning on whether it is “worthy” of publishing, whether journals will accept them, or whether others will understand them. In protecting these ideas as a means to protect ourselves from repeated rejection, we lose the ability to cultivate them.

## WHITENESS IN THE LIBRARY PROFESSION

According to the Medical Library Association (MLA) Diversity and Inclusion Task Force 2019 Survey Report, 5% of MLA members classified themselves as “Hispanic/Latinx.” An additional 6% of respondents self-identified as “Black or African American” and 6% as “Asian or Asian American” [4]. The 2017 American Library Association Demographic Study of its members cited 4.7% as self-identifying as “Hispanic or Latino,” 4.4% as “Black or African American,” 3.6% as “Asian,” 1.2% as “American Indian or Alaskan Native,” and .02% as “Hawaiian/Other Pacific Islander” [5].

In reflecting on these statistics, the fact is that Whiteness in LIS has always been and is still a fact of library life and can give the impression that librarianship is paralyzed by Whiteness [6]. The medical library profession's role in Whiteness, power, and privilege is marked by its own history of racism as well as its recent harmful mishandling of an editorial submitted by Black health sciences librarians that addresses anti-Blackness in publishing [7–10]. This is a prime example of why Latinx and BIPOC librarians often choose not to engage with scholarship for genuine concern of the trauma that comes with not understanding our lived experiences, perspectives, language, and critical frameworks in a White-dominated scholarly landscape.

## WHITENESS IN KNOWLEDGE PRODUCTION AND SCHOLARLY COMMUNICATION

It is important to acknowledge that Whiteness permeates every aspect of knowledge production: from gathering primary and secondary resources, to attributing theories and frameworks, to journal requirements, to, finally, the peer review and submission process. There are no avenues that exist absent of the ways in which scholarship is directly or indirectly informed by Whiteness. Calafell and Moreman, in a special issue that looked at performativity and *latinidad,* or the essence of Latinx, outlined specific trends in which scholarly communications impeded a BIPOC scholar's route to successfully publishing a work. This included the politics of using an auto-ethnographic voice, lack of sufficient peer reviewers with racial/cultural background and shared expertise, and perceptions of weak scholarly rigor when writing on topics related to race/ethnicity/intersectionality studies [11].

## BIAS IN PEER REVIEW AND INSTITUTIONAL HURDLES

Because publishing and academia consist of mostly White, middle-income, cisgender (identification with birth gender), heterosexual people, they are naturally biased toward the standards set by Whiteness, which manifests the Othering of non-White experiences including the histories and burdens of BIPOC. Marginalizing those outside this standard creates an awareness gap that is detrimental to equitable and culturally aware approaches in scholarly publishing. The term “peer review” is sorely misrepresentative, as BIPOC scholars cannot and do not feel that these reviewers are their true peers in the more comprehensive understanding of the meaning of being a peer or a member of a professional community. The limited demographic studies done on librarians specifically demonstrate the small (and in some cases a relative dwindling) number of BIPOC librarians and information professionals [12].

## LACK OF RESOURCES AND SUPPORT

Most health information professionals and health sciences librarians report acquiring important professional competencies through MLA programming, on-the-job training, and mentoring [13]. With Hispanic/Latinx information professionals and librarians being one of the smallest groups represented in academic libraries [14], our colleagues can often experience hurdles in securing appropriate professional development funding and on-the-job training, with mentoring relationships being specifically difficult to obtain in many circumstances, especially if the mentee is the only Latinx or BIPOC employed at the organization. In many instances, financial or any other kind of resources are lacking, and we are left not knowing what we can do to continue with our scholarship and professional growth. Even if there are resources available, we might not know how to navigate the hurdles, and there is little or no guidance on what to do.

## EXPLORING SOLUTIONS AND CALL TO ACTION

As Dr. Esther Choo observed in her John P. McGovern Award Lecture at the MLA '20 vConference, it is best to move away from “admiring the problem” and strive toward exploring solutions [15]. Therefore, viable solutions to these issues that can empower Latinx and BIPOC to have their voices heard, supported, and cultivated include:

- Peer mentoring among BIPOC colleagues
- Facilitating writing/publishing workshops
- Cross-promoting publishing opportunities on affinity caucus listservs
- Garnering support from White colleagues to proactively work alongside each other
- Implementing long-term institutional change in equitable scholarly practices

The professional organizations that BIPOC library workers support with their membership dues and conference registration fees also have a role in supporting them in return. The same holds true for White colleagues who benefit from the labor of BIPOC library workers in their shared professional world.

## CALL TO ACTION FOR LATINX LIBRARIANS

*Yo no soy marinero*

*Yo no soy marinero, soy capitán,*

*Soy capitán, soy capitán*

—La Bamba

The origins of the popular song “La Bamba,” most recently popularized by Chicano musicians Ritchie Valens in the 1950s and Los Lobos in the 1980s, come from 400 years ago [16]. This song survived in its creative and iterative forms through the centuries by those who continued to sing it from Veracruz to East Los Angeles and the world over [17]. Like the lyrics of “La Bamba,” we are not merely sailors of our *scholarly*ship, *somos capitán* (we are the captain)*.* As Latinx librarians, we are our best storytellers, in full control of our scholarly *testimonios* and narratives.

*Ya basta!* (Enough is enough!) Our voices need to be heard! Our stories need to be told! These barriers need to be addressed! If they persist, *nuestra voz e ideas* (our voices and ideas), *tu voz e ideas* (your voice and ideas) will not come to light. We, alone, do not own this work, and yet we also cannot wait for others to do this for us. We need to act, be heard, and advocate for one another. The best way for us to continue these efforts is to put pen to paper, *o manos al teclado* (hands to the keyboard), and manifest the scholarship and research endeavors we want to see.

Finally, full representation requires a top-down approach aimed at uplifting future Latinx leaders and mentoring of new Latinx librarians to inspire them to continue the cycle of peer-to-peer support. We need to build a community of practice to support fellow Latinx and BIPOC scholars and engage with each other's work. And in the process, we hope these efforts will work to dismantle White, heteronormative standards of traditional approaches to research and scholarship. Lastly, Latinx librarians need not forget the necessity of rediscovering *nuestra voz* (our voice) to carry our contributions steadfast and beyond the horizon.
